# A Novel EPO Receptor Agonist Improves Glucose Tolerance via Glucose Uptake in Skeletal Muscle in a Mouse Model of Diabetes

**DOI:** 10.1155/2011/910159

**Published:** 2011-06-30

**Authors:** Michael S. Scully, Tatiana A. Ort, Ian E. James, Peter J. Bugelski, Dorie A. Makropoulos, Heather A. Deutsch, Elsbet J. Pieterman, Anita M. van den Hoek, Louis M. Havekes, William H. duBell, Joshua D. Wertheimer, Kristen M. Picha

**Affiliations:** ^1^Discovery Research, Centocor R&D Inc., 145 King of Prussia Road, Radnor, PA 19087, USA; ^2^Trevena, Inc., Department of Biology, 1018 West 8th Avenue, King of Prussia, PA 19406, USA; ^3^TNO Biosciences, 2301 CE, Leiden, The Netherlands

## Abstract

Patients treated with recombinant human Epo demonstrate an improvement in insulin sensitivity. We aimed to investigate whether CNTO 530, a novel Epo receptor agonist, could affect glucose tolerance and insulin sensitivity. A single administration of CNTO 530 significantly and dose-dependently reduced the area under the curve in a glucose tolerance test in diet-induced obese and diabetic mice after 14, 21, and 28 days. HOMA analysis suggested an improvement in insulin sensitivity, and this effect was confirmed by a hyperinsulinemic-euglycemic clamp. Uptake of ^14^C-2-deoxy-D-glucose indicated that animals dosed with CNTO 530 transported more glucose into skeletal muscle and heart relative to control animals. In conclusion, CNTO530 has a profound effect on glucose tolerance in insulin-resistant rodents likely because of improving peripheral insulin sensitivity. This effect was observed with epoetin-*α* and darbepoetin-*α*, suggesting this is a class effect, but the effect with these compounds relative to CNTO530 was decreased in duration and magnitude.

## 1. Introduction

Erythropoietin (Epo) is a glycoprotein hormone secreted by the kidney and liver into circulation in response to hypoxia [1]. The principal function attributed to Epo is the regulation of red blood cell production, mediated by its specific cell surface receptor (EpoR) [2]. To this end, recombinant Epo forms (epoetin-*α*, epoetin-*β*, and the long-acting analogue darbepoetin-*α*) have been used to treat anemia in chronic kidney disease and chemotherapy-induced anemia in cancer patients [3]. However, EpoR expression in nonerythroid cells has raised questions as to additional biological functions of Epo in nonhaematopoietic tissues. Recent data suggest that endogenous Epo-EpoR signaling contributes to wound healing responses, physiological and pathological angiogenesis, and the body's innate response to injury in the brain and heart [4]. 

Several clinical studies have reported the effect of recombinant Epo on glucose metabolism in patients undergoing hemodialysis. Significant improvements in glucose utilization, mainly due to attenuation of insulin resistance were observed in 20 hemodialysis patients after three months of Epo therapy [5]. Tuzcu et al. found that patients with end-stage renal disease treated with Epo had a decreased HOMA index compared to untreated patients, suggesting an improvement in insulin sensitivity [6]. Glucose and lipid metabolism were studied in seven patients with end-stage renal disease being treated with erythropoietin to correct anemia [7]. This study showed that Epo alleviated insulin insensitivity and corrected lipid abnormalities in these patients. 

**Figure 1:**
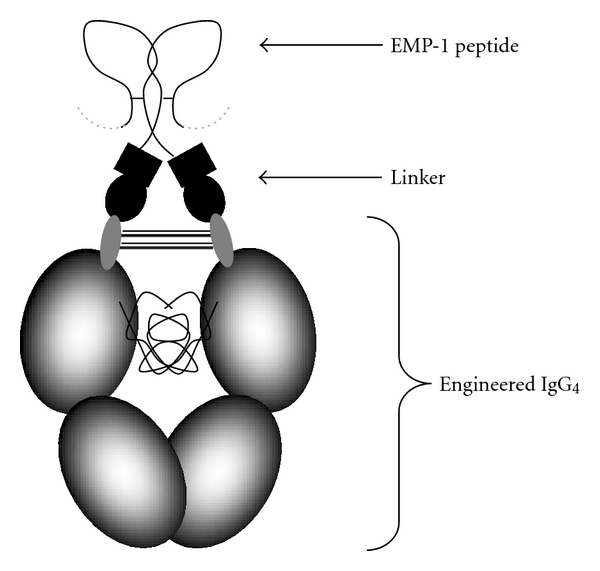
Schematic of CNTO 530. The schematic outlines the structure of CNTO 530 including the engineered Fc (CH2 and CH3 domains), a linker containing a partial VH region, and two EMP-1 peptides.

CNTO 530 is a MIMETIBODY that incorporates an erythropoietin mimetic peptide (EMP-1) genetically fused to a domain that includes the Fc portion of a human antibody (Figure 1). EMP-1 is a 20 amino acid peptide that binds and activates the Epo receptor [8, 9]. Significant engineering efforts in designing CNTO 530 resulted in a molecule where divalent display of EMP-1 peptides allows for the peptides to associate through a covalent interaction and activate the EpoR. The terminal half-life of CNTO 530 is sufficiently longer than the EMP-1 peptide in rodents [10] (40 hrs versus 4 hrs) which enables sustained activation of the EpoR. [10, 11]. As anticipated, this translates to a sustainable pharmacodynamic profile in mice. A single subcutaneous dose of CNTO 530 caused a long-lived stimulation of erythropoiesis that translated to increased hemoglobin values that were maintained for more than three weeks [10].

Here we report that CNTO 530, epoetin-*α*, and darbepoetin-*α* dose-dependently improved glucose tolerance in DIO mice. A hyperinsulinemic-euglycemic clamp study showed a beneficial effect of CNTO 530 in improving insulin sensitivity in diabetic mice. This effect was at least partially mediated via stimulation of insulin-dependent glucose transport into skeletal muscle. To our knowledge, this is the first time an increase in insulin-dependent glucose utilization in skeletal muscle has been demonstrated for an Epo receptor agonist.

## 2. Materials and Methods

### 2.1. Mice

All animal studies were performed according to the National Research Council's* Guide for the Care and Use of Laboratory Animals* and were approved by an internal IACUC. C57Bl/6J mice were maintained on a diet containing 60.9% kcal from fat (Purina TestDiets 58126, Richmond, IN) beginning at 4 weeks of age and all animals used in the DIO studies achieved three consecutive weeks of fasting blood glucose levels above 120 mg/dL. Lean littermates were maintained on a normal chow diet (Purina TestDiets 5010). Studies were completed when animals were 16–20 weeks of age.

### 2.2. Intraperitoneal Glucose Tolerance Test

Male DIO mice were randomized based on fasting blood glucose (FBG) following a 16-hour fast. Mice were dosed IV with phosphate-buffered saline (PBS), CNTO 530, epoetin-*α*, darbepoetin*α*, or CNTO1996 (a negative control protein identical to CNTO 530 but lacking the EMP-1 peptides). On the day of the glucose tolerance test, mice were given an intraperitoneal injection (1 mg/g) of glucose (Sigma). Blood glucose measurements were made 15, 30, 60, 90, 120, and 180 minutes following dosing using a hand-held glucometer (OneTouch Basic, LifeScan, Skilman, NJ).

### 2.3. Hematology and Insulin Measurements

Blood was collected into EDTA-treated tubes via a cardiac puncture from euthanized mice. Hematological analysis was completed immediately using an ADVIA 120 Analyzer (Siemens Medical Solutions Diagnostics, Tarrytown, NY). An additional aliquot of blood was centrifuged, and plasma was isolated. Plasma samples were frozen at –80°C until analysis by insulin ELISA (Crystal Chem Inc., Downers Grove, IL).

### 2.4. Conversion of mg/kg Doses with Epo-R Agonists to U/kg

Equation (1) was used to calculate UT-7 rHuEPO equivalents:

(1) $$\text{UT-}7\;\text{Units}/\mu \text{g}=\left(\text{Mol}\;\text{wt}\;\text{of}\;\text{ERA}\right)*\frac{\text{C}}{\left({\text{EC}}_{50}\;\text{for}\;\text{ERA}\right)},$$

where

(2) $$\text{C}=\left(120\;\text{Units}/\mu \text{g}\right)*\frac{\left({\text{EC}}_{50}\;\text{for}\;\text{rHuEPO}\right)}{34\;\text{kD}}.$$

And EC_50_ for ERA is 50% maximally effective concentration for an erythropoietin receptor agonist (molar concentration) in the UT7 viability assay [10].

Therefore, the doses in mg/kg were converted to U/kg by multiplying the respective dose (mg/kg) by the *in vitro* activity of each compound (epoetin-*α* = 120 U/*μ*g; darbepoetin-*α* = 199 U/*μ*g; CNTO 530 = 30 U/*μ*g).

### 2.5. Homeostasis Model Assessment (HOMA) Analysis

DIO mice were fasted overnight and randomized based on FBG and body weight. Mice were injected IV with PBS or CNTO 530 (0.3 mg/kg). Fourteen days after dosing, mice were fasted overnight (16 hrs) and blood was collected into tubes treated with EDTA via cardiac puncture. Fasting glucose levels were measured using a glucose oxidase reagent (Thermo Electron Corporation, Waltham, MA) according to the manufacturer's instructions. Fasting insulin levels were measured using the Mouse Ultra-Sensitive Insulin ELISA Kit (Crystal Chem Inc., Downers Grove, IL) according to the manufacturer's instructions. The HOMA index was calculated using (3) [12]:

(3) $$\begin{array}{cc} & \text{HOMA}=\\ & \;\frac{\left[\text{fasting}\;\text{insulin}\;\left(\text{mIU}/\text{L}\right)\;*\;\text{fasting}\;\text{glucose}\;\left(\text{mmol}/\text{L}\right)\right]}{22.5}. \end{array}$$

### 2.6. Validation of Glucometer Values

The studies reported here monitor the effect of CNTO 530 on glucose metabolism. CNTO 530 is an EpoR agonist that increases hematocrit (Hct) levels in a sustainable manner. It was previously reported that plethoric Hct values can influence the results obtained with a glucometer [13]. Many of the hand-held glucometer devices contain warnings that extreme hematocrit values can cause false glucose readings. To address the concern that the observed glucose levels could be the result of an artifact, a study comparing blood glucose measurements was completed using mice with elevated Hct levels. DIO mice were dosed with either CNTO 530 (0.3 mg/kg) or PBS, and whole blood and plasma were isolated fourteen days later after an overnight fast. Blood glucose levels were measured using a hand-held glucometer and with a VetAce Clinical Chemistry System (Alfa Wassermann Inc.) while plasma glucose was measured using a hand-held glucometer (OneTouch Basic, LifeScan), a VetAce instrument, and a glucose oxidase assay. CNTO 530-treated animals consistently decreased blood and plasma glucose level relative to control mice independent of the measurement method used (data not shown).

### 2.7. Hyperinsulinemic-Euglycemic Clamp Studies

C57Bl/6J male mice (6 weeks of age) were maintained on a semisynthetic high-fat diet (purina test diet no. 58126) for 14 weeks. Prior to the start of the study, the animals were randomized into 6 groups (*n* = 12) by body weight, fasting blood glucose, and fasting insulin. All animals were dosed IV with CNTO 530 (0.03 or 0.3 mg/kg) or PBS under isoflurane anesthesia. The animals remained on the same high-fat diet until the hyperinsulinemic-euglycemic clamp analysis was performed. The three groups (PBS, 0.03 mg/kg, 0.3 mg/kg) were subjected to clamp analysis one day after dosing, and the remaining three groups (PBS, 0.03 mg/kg, 0.3 mg/kg) were clamped 14 days after dosing. Blood hemoglobin was measured on the day of clamp analysis. Body weight and food intake was obtained weekly. Glucose levels were measured using the Freestyle Blood Glucose Measurement System from Memo Medical Mail Organization (Amersfoort, The Netherlands) according to the manufacturer's instruction. Plasma insulin was measured using the Ultrasensitive Mouse Insulin ELISA kit (Mercodia, Uppsala, Sweden) according to the manufacturer's instruction.

The clamp analysis was performed as described previously [14]. Briefly, animals were fasted for 16 hrs, anesthetized with acepromazine (6.25 mg/kg), midazolam (6.25 mg/kg), and fentanyl (0.3125 mg/kg), and an infusion needle was placed in one of the tail veins. Basal rates of glucose turnover were determined by means of a primed (0.7 *μ*Ci), continuous (1.2 *μ*Ci/h) infusion of [^3^H]-glucose for 60 minutes. Blood samples to determine basal glucose turnover were taken 50 and 60 minutes after the start of the infusion. The hyperinsulinemic condition was started with a bolus (7.0 mU), followed by continuous infusion of insulin (Actrapid, Novo Nordisk, 11.7 mU/h) and [^3^H]-glucose. A variable infusion of 12.5% unlabeled D-glucose solution was applied to maintain euglycemia as measured from blood samples (every 10 min) taken by tail bleeding. During the clamp (70, 80, and 90 minutes after the start of the insulin infusion), blood samples were drawn to determine glucose concentration and dpm of [^3^H]-glucose. Plasma for [^3^H]-glucose determinations was deproteinized by TCA precipitation. For each sample, an aliquot of the supernatant was counted directly and another was dried to remove ^3^H2O. To assess glucose oxidation at basal and hyperinsulinemic state, ^3^H_2_O level in plasma was calculated as the difference between dried and undried samples. At 90 minutes, additional samples were collected to determine insulin concentration. To estimate insulin-stimulated glucose uptake in individual tissues, 2-deoxy-D-[^14^C] glucose was administered as a bolus (2 *μ*Ci) 40 minutes before the end of the clamp. After sacrifice, liver, muscle (upper hind leg and heart) and adipose tissue (visceral and epididymal) were removed, immediately frozen in liquid N_2_, and stored at −20°C until further analysis. Measurement of tissue-specific 2-deoxy-D-[^14^C]-glucose was used to determine glucose uptake. For this analysis, part of the muscle and adipose tissue sample was homogenized (~10% wet wt/vol) in H_2_O. The homogenate was boiled, and the supernatant was subjected to an ion-exchange column to separate 2-deoxy-D-[^14^C]-glucose-6-P from 2-deoxy-D-[^14^C]-glucose. Radioactivity in the 2-deoxy-D-[^14^C]-glucose-6-P fraction represented the glucose taken up by the tissue.

### 2.8. Clamp Data Analysis

Significance of difference of values obtained were calculated using a nonparametric test for independent samples, Mann-Whitney *U*-test, using the computer program SPSS.

## 3. Results and Discussion

### 3.1. Glucose Tolerance Test at Various Times following Dosing

**Figure 2:**
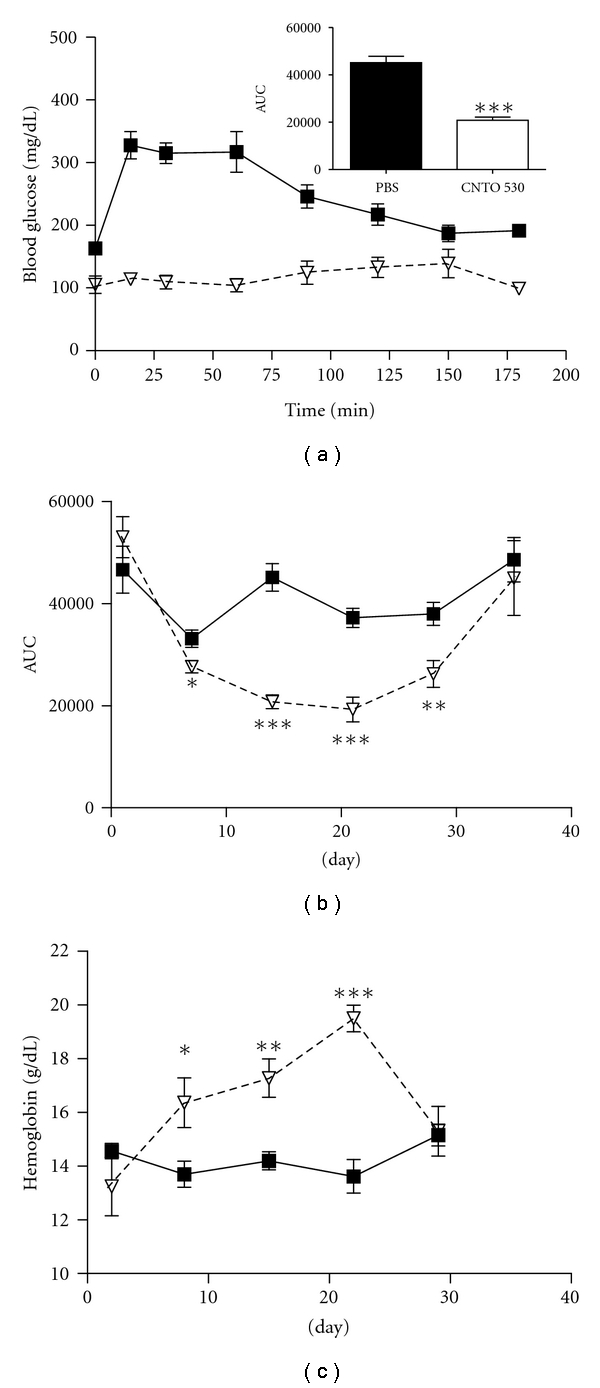
Single dose of CNTO 530 improves glucose tolerance and increases hemoglobin levels in DIO mice. (a) DIO mice (*n* = 7) were dosed IV with CNTO 530 (▿0.3 mg/kg) or PBS (■), and an IPGTT was done after 14 days. (b) IPGTT was done at various days following a single dose of CNTO 530 (0.3 mg/kg). The data were analyzed to determine the area under the curve (AUC) for CNTO 530-treated (▿) and PBS-treated (■) groups (*n* = 7). The AUC is plotted versus time. (c) The hemoglobin (g/dL) measured at various times following a single dose of CNTO 530 (0.3 mg/kg) was plotted versus time. The results are presented as mean ± SEM. *indicates a *P* value <  .05. **indicates a *P* value <.01. ***indicates a *P* value <  .005.

DIO mice were treated with a single IV dose of CNTO 530 (0.3 mg/kg), and glucose metabolism was monitored using an intraperitoneal glucose tolerance test (IPGTT) at various times following dosing (day 1 to 35). CNTO 530 did not influence glucose tolerance 24 hours after dosing. However, a significant improvement was observed in both fasting glucose (179 ± 29 versus 133 ± 19 mg/dL for untreated versus treated) and overall glucose tolerance seven days after a single dose of CNTO 530 (Figure 2(b)). In a separate study, the activity of CNTO 530 was compared to a control protein that lacked the EMP-1 peptides to ensure that the observed effects were due to the peptide and not the Fc. The glucose lowering effect was not observed in the mice treated with the protein lacking the EMP-1 peptides (data not shown). Fasting blood glucose remained improved in the treated animals 14 and 21 days after CNTO530 treatment, but was similar to the untreated group after 35 days (data not shown). The area under the curve (AUC) for the glucose tolerance tests showed a significant improvement with CNTO 530 treatment on days 7, 14, 20, and 28 (Figure 2(b)). The most dramatic effect on glucose tolerance following CNTO 530-treatment was observed on day 14 where glucose was cleared from circulation by the 15-minute time point (Figure 2(a)). CNTO 530-mediated acceleration of glucose utilization was diminished but still significant by day 28 and not seen by day 35 (Figure 2(b)). Hemoglobin levels followed a similar time-dependent change, showing an increase that peaked on day 22 and returned to normal levels by day 29 (Figure 2(c)).

Similar studies were completed in lean littermate mice fed a normal chow diet. Mice (*n* = 7) were given a single administration of CNTO 530 (0.01–0.3 mg/kg), and glucose tolerance tests were completed after 1 and 14 days. As we observed in the diabetic rodents, no significant changes were seen in glucose clearance 24 hours after dosing. However, there were significant improvements in the area under the curve in the glucose tolerance test in animals dosed with 0.3 and 0.1 mg/kg CNTO 530 as compared to PBS (22865.4 ± 1900.2, 24478.9 ± 1093.4, and 28773.2 ± 1368.3, resp., mean ± SEM). There were no dose-dependent changes in the fasting blood glucose.

### 3.2. Glucose Tolerance Test-Dose Titration

**Figure 3:**
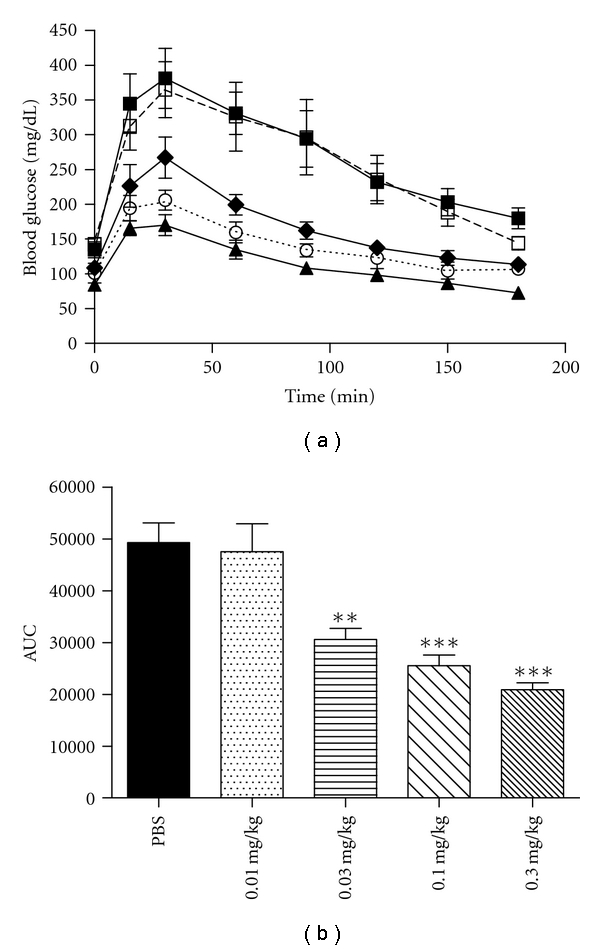
Dose response of CNTO 530 at day 14. PBS (■) or CNTO 530 was dosed IV at increasing concentrations (0.01□, 0.03♦, 0.1○, 0.3▲) in DIO mice (*n* = 5). (a) Fourteen days after dosing, an IPGTT was performed. (b) The AUC from part A was plotted for each dose of CNTO 530. The results are presented as mean ± SEM. **indicates a *P* value <  .01. ***indicates a *P* value <  .005.

To determine whether the effect of CNTO 530 on glucose tolerance was dose dependent, GTTs were repeated in DIO mice fourteen days after IV dosing with increasing concentrations of CNTO 530 (0.01, 0.03, 0.1, 0.3 mg/kg). A dose-dependent reduction in the AUC was observed with 0.03, 0.1, and 0.3 mg/kg whereas a dose of 0.01 mg/kg had no effect (Figures 3(a) and 3(b)).

### 3.3. Glucose Tolerance Test with Other EpoR Agonists

**Figure 4:**
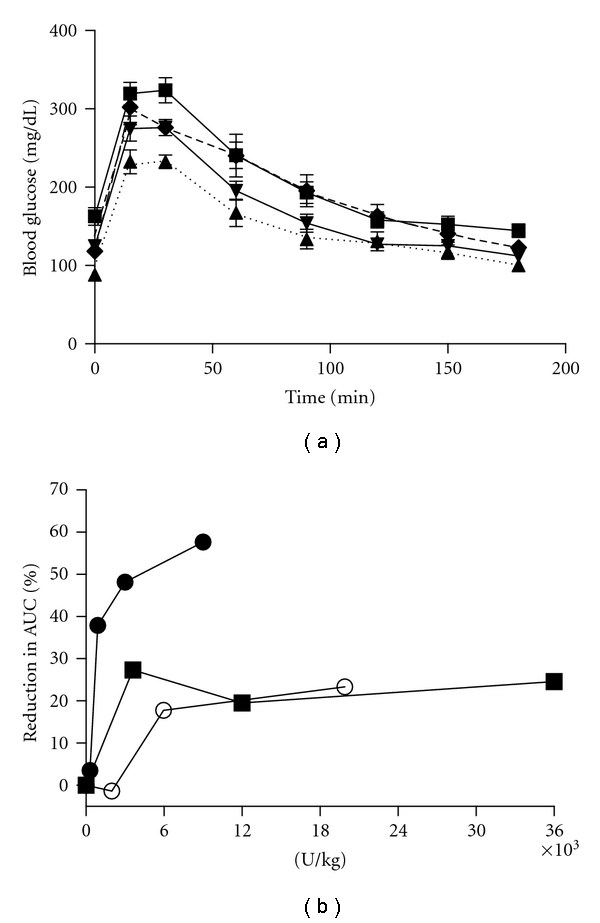
Dose response of epo receptor agonists. (a) Increasing concentrations of darbepoetin-*α* (0.03♦, 0.1▾, 0.3▲) or PBS (■) were dosed IV to DIO mice (*n* = 7). Seven days after dosing, an IPGTT was completed. (b) The same experiment described in A was completed with epoetin-*α* (*n* = 7) at day 5 *⬤*, darbepoetin (*n* = 7) at day 7 ■, and CNTO 530 (*n* = 5) at day 14 ○. The reduction in AUC was calculated for each molecule and was plotted versus the U/kg of Epo receptor agonist.

CNTO 530 is an agonist to the EpoR, yet there is no homology between the molecular structure of CNTO 530 and that of Epo itself. Therefore, there is the potential that either the EMP-1 peptide or the Fc domain of the molecule interacts with a different receptor entirely, thereby effecting glucose metabolism through a receptor other than the EpoR. To address this point, similar glucose tolerance studies were repeated with epoetin-*α* and darbepoetin-*α*. To compare the pharmacodynamic effects of different EpoR agonists, an equipotent dose of each molecule was used (see Section 2.4). In addition, the GTTs were conducted 5 and 7 days after dosing for epoetin-*α* and darbepoetin-*α*, respectively, given that this is the time when hemoglobin levels are maximally elevated in rodents [11]. Similar to CNTO 530, epoetin-*α* and darbepoetin-*α* increased glucose clearance in a dose-dependent manner (Figure 4(a) and data not shown). However, the maximal reduction in AUC during a GTT was not as profound relative to CNTO 530, suggesting that the extended pharmacokinetic profile observed with CNTO 530 contributes to an improved pharmacodynamic glucose effect (Figure 4(b)).

### 3.4. HOMA Analysis

**Figure 5:**
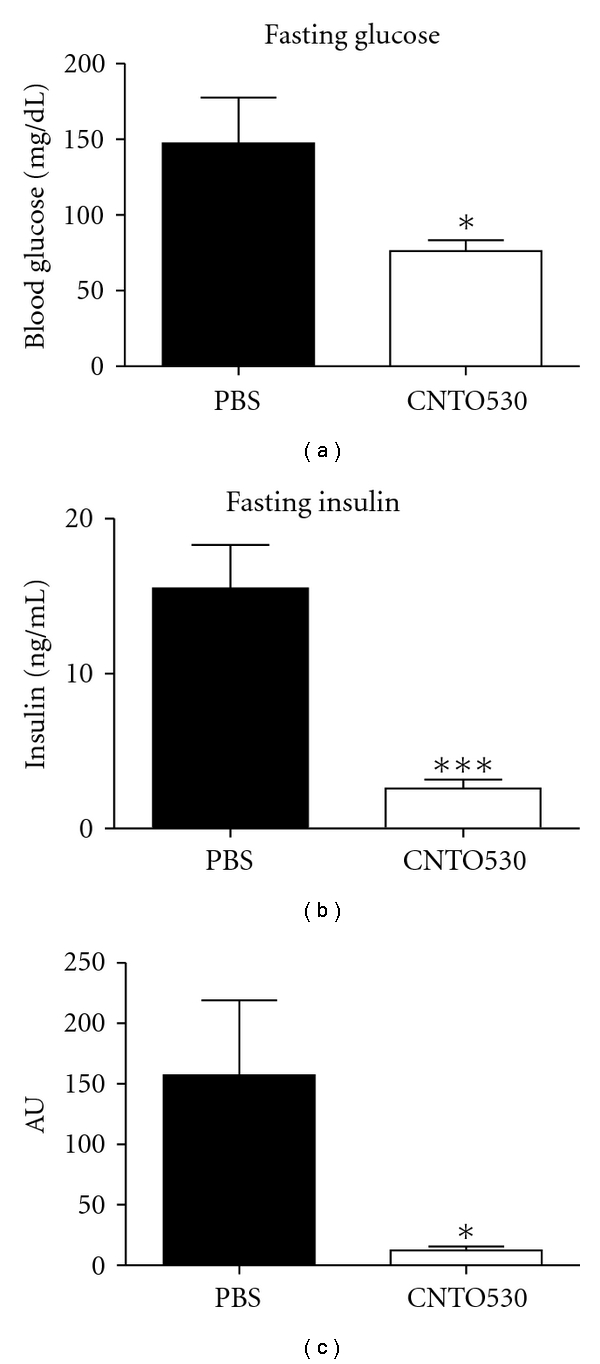
HOMA analysis following a single dose of CNTO 530. CNTO 530 (0.3 mg/kg) was dosed IV to DIO mice (*n* = 7). Fourteen days later, (a) FBG and (b) fasting insulin levels were measured. (c) HOMA analysis was calculated for each group. The results are presented as mean ± SEM. *indicates a *P* value <  .05. ***indicates a *P* value <  .005.

Homeostasis model assessment (HOMA) is a rather indirect way to assess insulin sensitivity, as it is less invasive and less time consuming relative to clamp analysis [12] HOMA was initially used to determine if there was a gross change in insulin sensitivity in DIO mice treated with CNTO 530. Fasting glucose and insulin levels in CNTO 530-treated mice were significantly reduced compared to the PBS-treated control 14 days after IV dosing (Figures 5(a) and 5(b)). As a result, CNTO 530 decreased the calculated HOMA index more than 10-fold relative to control animals, suggesting a significant improvement in insulin sensitivity (Figure 5(c)).

### 3.5. Hyperinsulinemic Clamp

To delineate the CNTO 530 mechanism of reducing blood glucose, we performed a hyperinsulinemic-euglycemic clamp in anesthetized DIO mice. C57/Bl6 mice were maintained on a high-fat diet for 14 weeks prior to the CNTO 530 treatment to induce obesity and insulin resistance (mean BW 45.7 ± 3.5 g, mean fasting insulin 3.8 ± 3.3 ng/mL, mean fasting blood glucose 120.6 ± 18 mg/dL). The clamp was performed one and fourteen days following a single IV dose of vehicle or CNTO 530 (0.03 and 0.3 mg/kg). 

In agreement with previous studies, no significant changes in hemoglobin or FBG were detected one day after CNTO 530 administration (Table 1). Fourteen days after treatment, however, both doses of CNTO 530 significantly increased hemoglobin (Table 1). CNTO 530 (0.3 mg/kg) significantly reduced fasting blood glucose fourteen days after treatment, consistent with the previous studies (Table 1). Administration of CNTO 530 did not alter body weight (Table 1) or food intake (data not shown). 

**Figure 6:**
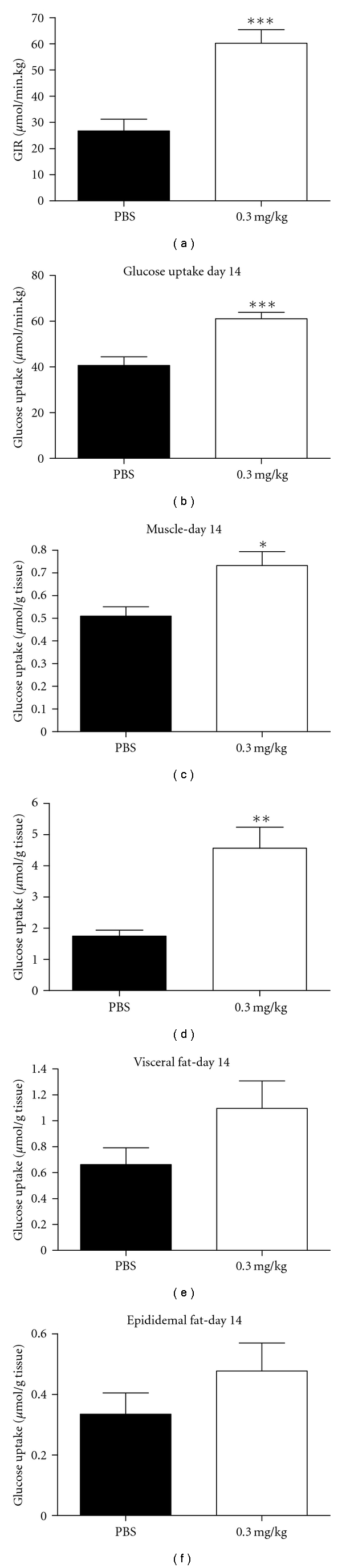
Hyperinsulinemic-euglycemic clamp following a single dose of CNTO 530. (a) The glucose infusion rate in DIO mice treated IV with CNTO 530 (0.3 mg/kg) or PBS measured fourteen days after drug administration (*n* = 12). (b) The glucose uptake into peripheral tissues in DIO mice treated IV with CNTO 530 (0.3 mg/kg) or PBS measured fourteen days after drug administration. A nonhydrolyzable, radiolabeled glucose molecule was dosed as a bolus to the animals 40 minutes before the end of the clamp. The amount of glucose taken up into the skeletal muscle (c), the heart (d), the visceral fat (e), and the epididymal fat (f). *indicates a *P* value <  .05. ***indicates a *P* value <  .005.

During the clamp studies, the circulating levels of glucose and the difference in plasma insulin levels were not statistically different between the groups (Table 1). The glucose infusion rate (GIR) required to maintain the target glucose concentration during the clamp study was not significantly different between the groups one day after CNTO 530 administration, although there was a tendency towards a higher GIR in animals treated with the high dose of CNTO 530 (0.3 mg/kg) relative to the control group (Table 1). However, GIR was significantly increased (2.2-fold) in DIO mice 14 days after administration of the 0.3 mg/kg dose of CNTO 530 (Figure 6(a), Table 1). Hepatic glucose production (HGP), measured by the isotope dilution of the infused [^3^H]-glucose, was not different between the treatment groups with the exception of an increased basal HGP detected in mice treated with CNTO 530 (0.3 mg/kg) one day after IV injection (Table 1). Consistent with GIR changes, the rate of whole-body glucose disappearance was significantly elevated in the CNTO 530 (0.3 mg/kg) groups by 40% and 50% after one and fourteen days after treatment, respectively (Table 1). Whole-body glucose oxidation did not change between the groups at basal or hyperinsulinemic state (Table 1). Insulin-stimulated glucose transport activity in skeletal muscle, heart, and adipose tissues was estimated by monitoring the rate of whole-body clearance of 2-deoxyglucose and its rate of phosphorylation during the last 40 minutes of the insulin clamp. Since 2-deoxyglucose is transported into tissue and is phosphorylated but not further metabolized, determination of the tissue content of 2-deoxyglucose-6-phosphate can be used to produce a valid estimate of the rate of glucose transport and phosphorylation in individual tissues. Hyperinsulinemic glucose transport activity in skeletal muscle and heart was significantly increased by approximately 30% and 50%, respectively, in CNTO 530-treated (0.3 mg/kg) mice compared to control mice 14 days after dosing (Figures 6(c) and 6(d), Table 1). CNTO 530 (0.3 mg/kg) administration resulted in increased insulin-stimulated glucose transport in visceral adipose by 25% and 64% after one and fourteen days of dosing respectively, but these differences did not achieve statistical significance (Figure 6(e), Table 1). No change in insulin-stimulated glucose transport was detected in epididymal adipose tissue between the groups (Figure 6(f), Table 1).

Clinical trial data have shown that patients whose anemia is treated with recombinant human Epo often exhibit improved peripheral insulin sensitivity, although the mechanism of action for this is unclear. For example, Mak [15] has suggested that the improvement in insulin sensitivity in dialysis patients treated with EPO is a direct effect of the correction of the anemia associated with kidney failure. Other authors suggest that erythropoietin increases the oxygen supply to tissues and have shown a strong correlation between pO_2_ and peripheral insulin sensitivity [16]. However, there are contradicting studies that suggest that the changes in insulin sensitivity are not directly linked to hematocrit levels or oxygen capacity. One such study monitored insulin sensitivity via an euglycemic insulin clamp and showed that patients treated with Epo exhibited improved insulin sensitivity and glucose clearance before hematocrit began to increase [17]. Another study suggested that Epo treatment increased insulin sensitivity by reducing iron stores and improving the chronic inflammatory state of dialysis patients [18].

The role of Epo signalling in glucose metabolism and insulin resistance has also been studied in murine models. In an STZ-model of diabetes in NOD-SCID mice, treatment with either rhEpo or CEPO had no effect on insulin sensitivity [19]. However, this may not be an appropriate animal model since most of the islets in the STZ-treated animals would be destroyed and insulin levels would likely be low. In a more relevant study, Epo receptor-null mice that were rescued with EpoR expression restricted to hematopoietic tissue developed obesity and glucose intolerance compared to control mice [20]. This study clearly suggests a role for Epo receptor signaling in nonhematopoietic tissues in regulating glucose metabolism and insulin sensitivity. Finally, several weeks of dosing epoetin-*α* resulted in improved glucose levels in multiple murine models type 2 diabetes including ob/ob mice and protein tyrosine phosphatase-1B (PTP-1B) knockout mice [21]. The sustained reduction in glucose levels also resulted in improvements in HbA1c. 

We initiated our studies in transgenic mice overexpressing human Epo. A metabolic phenotype was observed in these mice, as fasting blood glucose levels were markedly reduced relative to the wild-type controls (85.0 ± 11.4 (wt) versus 49.0 ± 7.7 (Epo transgenics) mg/dL, unpublished data). However, the extremely high hematocrit levels achieved in these mice (>80%) made it technically challenging to continue metabolic studies in these animals. Katz et al. were able to complete similar studies showing that tg6 mice overexpressing human EPO had reduced levels of glucose and improvements in HbA1c [21].

We chose instead to conduct a hyperinsulinemic-euglycemic clamp study in DIO mice. We monitored the effect of CNTO 530 on glucose uptake and hepatic glucose production in mice fed a high-fat diet for 14 weeks prior to treatment. In addition, we measured tissue-specific glucose uptake in skeletal and cardiac muscle, liver and adipose tissue. As expected, CNTO 530 increased hemoglobin and decreased fasting blood glucose fourteen days following IV administration. In addition, insulin sensitivity was also improved at this time, as indicated by a significant increase in the rate of glucose infusion required to maintain euglycemia in the CNTO 530-treated animals. Analysis of both hepatic glucose production and glucose uptake by peripheral tissues demonstrated that the improvement in insulin sensitivity was largely due to CNTO 530-mediated enhancement of insulin-stimulated glucose uptake in skeletal muscle and heart. CNTO 530 did not affect glucose oxidation suggesting that CNTO 530 may activate nonoxidative mechanisms of glucose utilization in skeletal muscle and heart. The effects of CNTO 530 on glucose metabolism were a result of Epo receptor signaling as similar improvements were observed in DIO mice treated with epoetin-*α* or darbepoetin-*α*. Epoetin-*α* or darbepoetin-*α* was dosed at levels that induced comparable increases in hematocrit relative to CNTO 530. Mice treated with these EpoR agonists also exhibited improved glucose tolerance but the time course of the effect was shorter and the increase in glucose tolerance was not nearly as profound or long-lived as that observed with CNTO 530. 

The mechanism by which Epo receptor agonists improve glucose metabolism is still unclear in both rodents and humans. Unfortunately, our data does not help to clarify whether the effect of Epo receptor agonists on glucose metabolism is linked solely to hematopoiesis. For example, there is a significant increase in hemoglobin in response to a 0.3 mg/kg dose of CNTO 530 at day 7 (Figure 2(c)) but minimal effect on glucose tolerance (Figure 2(b)) and the glucose tolerance effect persists through day 28 (Figure 2(b)), at which time the hemoglobin is back to baseline (Figure 2(c)). In addition, while epoetin-*α* and darbepoetin-*α* increased hemoglobin similar to CNTO 530 (day 5 CNTO 530 0.3 mg/kg Hct 60%, Epo 0.1 mg/kg Hct 58%, Darbe 0.03 mg/kg Hct 58%), they had a reduced effect on glucose tolerance (Figure 4(b)). 

One possible mechanism for the improved glucose tolerance is Epo receptor signaling in striated (skeletal and cardiac) muscle. In our study, skeletal and cardiac muscle each showed increased glucose uptake during the clamp study (Table 1). Both skeletal [22] and cardiac [23] muscle have been shown by immunohistochemistry to express sarcolemmal Epo receptors. We confirmed the presence of the receptor on rat cardiomyocytes by confocal microscopy and used RT-PCR to show expression of EpoR mRNA in skeletal muscle of DIO mice (data not shown). Thus CNTO 530 may interact directly with sarcolemmal Epo receptors and induce a signaling cascade that increases insulin sensitivity and glucose utilization in these tissues.

Other studies support a role for skeletal muscle Epo receptor signaling in the metabolic effects of Epo administration. A recent study demonstrated that mice overexpressing murine Epo were protected against diet-induced obesity [24]. For this study, doxycycline-dependent transgenic Epo over-expression was established in the right tibialis cranialis muscle in mice. Both Epo-expressing and contralateral muscles from these mice exhibited hypertrophy and increased vascularization. Further, the muscles had increased capacity to oxidize palmitate compared to control muscles. In another study, muscle-specific transgenic expression of Akt1 resulted in a phenotype which included muscle fiber hypertrophy, resistance to diet-induced obesity and improved glucose tolerance [25]. EpoR signaling activates Akt through STAT5 and PI3 kinase [26] so one possible explanation for our data is that CNTO 530 is activating Akt1 in skeletal muscle, and thus improving glucose utilization. In this regard, PI3 kinase/Akt activation is an essential step for insulin-induced GLUT4 translocation and subsequent glucose uptake in skeletal muscle [27].

## 4. Conclusion

In conclusion, the present study demonstrates that administration of a single dose of the novel Epo receptor agonist CNTO 530 to diet-induced obese mice results in improved glucose tolerance and insulin sensitivity. The increased glucose tolerance resulted at least in part from increased uptake of glucose by skeletal and cardiac muscle. The molecular mechanism responsible for translating Epo receptor signaling into improved glucose tolerance remains elusive. However, CNTO 530 could represent a novel therapeutic mechanism of type 2 diabetes, providing that the drug can be dosed at a level that provides the beneficial metabolic effects without a risk of substantially increasing hematocrit.

## Figures and Tables

**Table 1:** Hyperinsulenemic euglycemic clamp characteristics in DIO mice treated with single IV administration of CNTO 530 (0.03 mg/kg and 0.3 mg/kg). Insulin clamp was performed at one and fourteen days after drug administration. The results are presented as mean ± SEM. ^#^indicates a *P* value <  .05 relative to control group.

	Day 1			Day 14		
	PBS	0.03 mg/kg	0.3 mg/kg	PBS	0.03 mg/kg	0.3 mg/kg
Body weight, g	45.7 ± 1.5	46.6 ± 1.7	44.7 ± 1.8	46.9 ± 1.5	43 ± 4.8	42.9 ± 4.8
Hemoglobin, mmol/L	8.6 ± 0.3	8.4 ± 0.5	8.4 ± 0.3	9.3 ± 0.3	10.0 ± 0.2^#^	11.3 ± 0.2^#^
Fasting glucose, mmol/L	5.5 ± 0.3	5.4 ± 0.4	5.5 ± 0.6	4.7 ± 0.4	4.5 ± 0.3	2.8 ± 0.15^#^
Fasting plasma insulin, ng/mL	1.9 ± 0.3	2.4 ± 0.5	2.1 ± 0.4	1.8 ± 0.2	2.1 ± 0.4	1.8 ± 0.3
Fasting HGP, *μ*moL/min.kg	29.5 ± 0.6	39.1 ± 6.3	36.3 ± 2.3^#^	41.6 ± 3.6	47.1 ± 4.4	38.5 ± 2.8
Fasting glucose oxidation, dpm/*μ*L	65 ± 9	52 ± 12	55 ± 9	85 ± 7	86 ± 4	70 ± 5
GIR, *μ*moL/min.kg	26.3 ± 2.6	26.6 ± 4.1	36.9 ± 4.2	26.7 ± 4.5	33.9 ± 3.9	60.2 ± 5.2^#^
Plasma insulin during hyperinsulinemic clamp, ng/mL	9.1 ± 0.6	10.9 ± 1.5	8.6 ± 0.6	12.2 ± 0.7	12.4 ± 1.2	14.3 ± 1.6
Hyperinsulinemic glucose uptake, *μ*moL/min.kg	28.4 ± 1.9	32.1 ± 3.2	39.9 ± 2.8^#^	40.7 ± 3.7	46.4 ± 2.8	61.1 ± 2.7^#^
Hyperinsulinemic glucose production, *μ*moL/min.kg	3.6 ± 1.6	6.02 ± 2.8	2.7 ± 3	13.9 ± 1.2	12.8 ± 1.6	0.96 ± 5.0
Hyperinsulinemic glucose oxidation*, dpm/*μ*L	184 ± 14	181 ± 10	187 ± 12	219 ± 15	192 ± 8.5	203 ± 13
Skeletal muscle glucose uptake, *μ*moL/g tissue	0.53 ± 0.1	0.50 ± 0.10	0.56± 0.05	0.51 ± 0.04	0.48 ± 0.04	0.73 ± 0.06^#^
Heart glucose uptake, *μ*moL/g tissue	3.0 ± 0.6	4.4 ± 1.3	5.3 ± 1.6	1.8 ± 0.2	2.2 ± 0.2	4.6 ± 0.7*
Visceral adipose glucose uptake, *μ*moL/g tissue	0.55 ± 0.06	0.49 ± 0.07	0.74 ± 0.08	0.67 ± 0.13	0.50 ± 0.21	1.1 ± 0.2
Epididemal adipose glucose uptake, *μ*moL/g tissue	0.47 ± 0.1	0.38 ± 0.08	0.51 ± 0.06	0.33 ± 0.02	0.43 ± 0.07	0.48 ± 0.09

*Measured 2 h after clamp initiation.

## Abbreviations

DIO: Diet-induced obese
Epo: Erythropoietin
AUC: Area under the curve
GTT: Glucose tolerance test
Hct: Hematocrit
GIR: Glucose infusion rate
PBS: Phosphate-buffered saline.
